# Comprehensive analysis of the mechanism and treatment significance of Mucins in lung cancer

**DOI:** 10.1186/s13046-020-01662-3

**Published:** 2020-08-17

**Authors:** Yue Ning, Hongmei Zheng, Yuting Zhan, Sile Liu, Yang yang, Hongjing Zang, Jiadi Luo, Qiuyuan Wen, Songqing Fan

**Affiliations:** Department of Pathology, The Second Xiangya Hospital, Central South University, Changsha, 410011 Hunan China

**Keywords:** Lung cancer, Mucins, Biomarkers, Treatment of lung cancer

## Abstract

Aberrant expression of mucin proteins has played a complex and essential role in cancer development and metastasis. Members of the mucin family have been intimately implicated in lung cancer progression, metastasis, survival and chemo-resistance. During the progression of lung cancer, mucin proteins have involved all of the procession of lung cancer, which is interacted with many receptor tyrosine kinases signal pathways and mediated cell signals for tumor cell growth and survival. Mucins thus have been considerable as the indicator of negative prognosis and desirable therapeutic targets of lung cancers. In this review, we comprehensively analyzed the role of each member of the mucin family in lung cancer by combining open-accessed database analysis and assembling cutting-edge information about these molecules.

## Background

Lung cancer has ranked the most common cause of cancer death worldwide. Every year, there are about 1.8 million people being diagnosed with lung cancer, and 1.6 million people died from the disease [1]. Approximately 85% of patients had a group of histological subtypes collectively known as non-small cell lung cancer (NSCLC), in which lung adenocarcinoma (LUAD) and lung squamous cell carcinoma (LUSC) have been the most common subtypes [2]. Recently significant advancement has been made in the driver genes research, screening biomarkers, and personalized therapy (precision medicine) of lung cancer, the 5-year relative survival rate for lung cancer has been 19% overall (16% for men and 23% for women); 24% for non-small cell; and 6% for small cell tumors [3]. However, there still remain several challenges as following: we need to identify new driver gene alterations to expand the population benefited from targeted therapies; It is important to understand the mechanisms responsible for resistance to targeted therapy for further prevention or overcoming; also better predictors of responses to immunotherapy, new drugs and rationally designed drug combination therapies need to screen [4].

Mucins are classified into two major categories depended on their structure-membrane mucins and secreted mucins. The membrane mucins are consisted of eleven members as MUC1, MUC3A, MUC3B, MUC4, MUC12, MUC13, MUC15, MUC16, MUC17, MUC20 and MUC21; while secreted mucins are comprised of seven members which can be further subdivided into gel-forming mucins (MUC2, MUC5AC, MUC5B, MUC6, MUC19) and non-gel-forming mucins (MUC7, MUC8). All mucin members have at least one mucin-like domain which contains a high proportion of tandem repetitive structures of prolines, threonines and serins (which form the PTS domain). And the PTS domain of the mucins is extensively glycosylated at the threonine and serine residues through GalNAc O-linkages. The two kinds of mucins have different functions in the human body. The membrane mucins are located in the ductal surfaces of organs epithelial cells served as a physical barrier. The transmembrane mucins are primarily located on the apical membrane of epithelial cells, where they could play a role in cell signaling. They all protect the integrity of epithelial cells from different environmental stresses. For example, they could degraded enzymes by forming a physical, chemical and immunological barrier and interact with many receptor tyrosine kinases mediated cell signals [5]. Any alteration of MUCs expression or glycosylation pattern will significantly affect tumor cell growth, differentiation and survival which enabled them regard as potent cancer-inducing molecules [5–7].

In this review, we have drawn an overview of the mucin family and discussed the role of each mucin members in tumorigenesis and metastasis and recent advances in tumor research. We will concentrate on the importance of mucin proteins on cellular signaling pathways and its role in targets and immune therapy of lung cancer.

### Expression and mutation landscape of Mucins in NSCLC

**Fig. 1:**
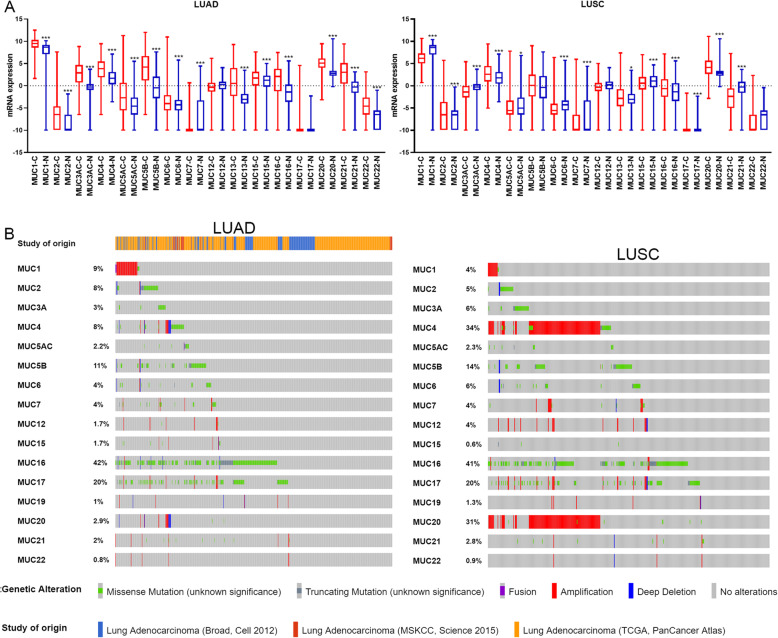
Mucins expression and mutation in non-small cell lung cancer. **a**. mRNA expression of mucin in lung adenocarcinoma and lung squamous cell carcinoma. **b**. mutation rate of mucins in in lung adenocarcinoma and lung squamous cell carcinoma. ^*^P < 0.05, ^**^P < 0.01, ^***^P < 0.0001

TCGA-GTEx mixed data Cohort, which contained 1410 lung cancer and normal lung tissue samples was downloaded from UCSC Xena [8–10] to analyze Mucins expression (Fig. 1a). According to our analysis, we found that MUC1, MUC2, MUC3AC, MUC4, MUC5AC, MUC5B, MUC6, MUC13, MUC15, MUC16, MUC20, MUC21, MUC22 elevated than normal lung tissue while MUC7 decreased in LUAD. However, in LUSC, MUC1, MUC3AC, MUC5AC, MUC6, MUC7, MUC15, MUC17, MUC21 indicated lower expression compared with normal lung tissue, while MUC4, MUC13, MUC16, MUC20 increased. These results somewhat have contradicted with the existing research that each of MUC1 and MUC5AC had a high protein expression in lung carcinoma. Lappi-Blanco et al. summarized the MUC1 expression in lung cancer, which found high expression of MUC1 predicts poor survival in the majority of studies [11]. Especially, Guddo et al. and Woenckhaus et al. demonstrated MUC1 expression was associated with poor prognosis in squamous cell cancers patients [12, 13]. Considering to MUC5AC, Yu et al. identified MUC5AC overexpressed in stage I/II NSCLC patients [14, 15]. In addition, both MUC1 and MUC5AC have higher expression in adenocarcinomas compared with squamous cell carcinomas [14, 16]. However, our analysis based on public databases indicated that both MUC1 and MUC5AC mRNA overexpressed in LUAD, but decreased in LUSC. Hence, it is necessary to study the function of Mucins in LUAD and LUSC separately.

The mutation analysis based on the cBioportal [17, 18] had performed in Lung Adenocarcinoma (Broad, Cell 2012), Lung Adenocarcinoma (MSKCC, Science 2015), Lung Adenocarcinoma (TCGA, PanCancer Atlas) and Lung Squamous Cell Carcinoma (TCGA, PanCancer Atlas). 784 LUAD samples and 469 LUSC samples were incorporated. The results demonstrated that MUC4 (34% Amplication), MUC16 (41%), MUC17 (20% Missense), MUC20 (31% Amplication), MUC5B (14% Missense) existed high mutation rates in LUSC patients while in LUAD patients there had been high mutation in MUC5B (11% Missense), MUC16 (42% Missense), MUC17 (20% Missense). In addition, MUC16 and MUC17 have been the top 10 mutated genes of LUAD, and MUC16, MUC17 and MUC5B have been the top 50 mutated genes in LUSC (Fig. 1b). It has recently reported that co-occurring genomic alterations as mediators of diverse NSCLC phenotypes impacted molecular stratification framework shave, which emerged as a major tenets of the molecular diversity of NSCLC [19]. Therefore, we assessed the relationship between MUC mutation (Table.1). In LUAD, MUC5B were co-occurrence with MUC2, MUC6, MUC16, and MUC17; MUC16 existed co-occurrence with MUC2, MUC3A; MUC17 had co-occurrence with MUC2 and MUC12. In LUSC, MUC4 had co-occurrence with MUC20, MUC12 had co-occurrence with MUC17; and MUC21 had co-occurrence with MUC22.

**Table 1:** Co-occurrence analysis of Mucins in lung cancer

Cancer	A	B	Neither	A Not B	B Not A	Both	Log2 Odds Ratio	***p***-Value	q-Value	Tendency
**LUAD**	MUC2	MUC16	404	21	265	38	1.464	< 0.001	0.003	Co-occurrence
	MUC3A	MUC16	421	4	284	19	2.816	< 0.001	< 0.001	Co-occurrence
	MUC5B	MUC16	395	30	244	59	1.671	< 0.001	< 0.001	Co-occurrence
	MUC12	MUC17	580	0	137	11	> 3	< 0.001	< 0.001	Co-occurrence
	MUC16	MUC17	368	212	57	91	1.471	< 0.001	< 0.001	Co-occurrence
	MUC2	MUC17	542	38	127	21	1.238	0.003	0.032	Co-occurrence
	MUC5B	MUC17	521	59	118	30	1.167	0.001	0.013	Co-occurrence
	MUC3A	MUC20	687	19	18	4	> 3	0.004	0.035	Co-occurrence
	MUC4	MUC20	663	43	1	21	> 3	< 0.001	< 0.001	Co-occurrence
	MUC21	MUC22	712	10	0	6	> 3	< 0.001	< 0.001	Co-occurrence
	MUC2	MUC5B	599	40	70	19	2.023	< 0.001	< 0.001	Co-occurrence
	MUC5B	MUC6	620	79	19	10	2.046	0.001	0.014	Co-occurrence
	MUC3A	MUC7	681	14	24	9	> 3	< 0.001	< 0.001	Co-occurrence
**LUSC**	MUC4	MUC20	306	18	5	140	> 3	< 0.001	< 0.001	Co-occurrence
	MUC12	MUC17	373	0	76	20	> 3	< 0.001	< 0.001	Co-occurrence
	MUC21	MUC22	456	9	0	4	> 3	< 0.001	< 0.001	Co-occurrence

### Effects of Mucins on cellular signaling pathways

**Fig. 2:**
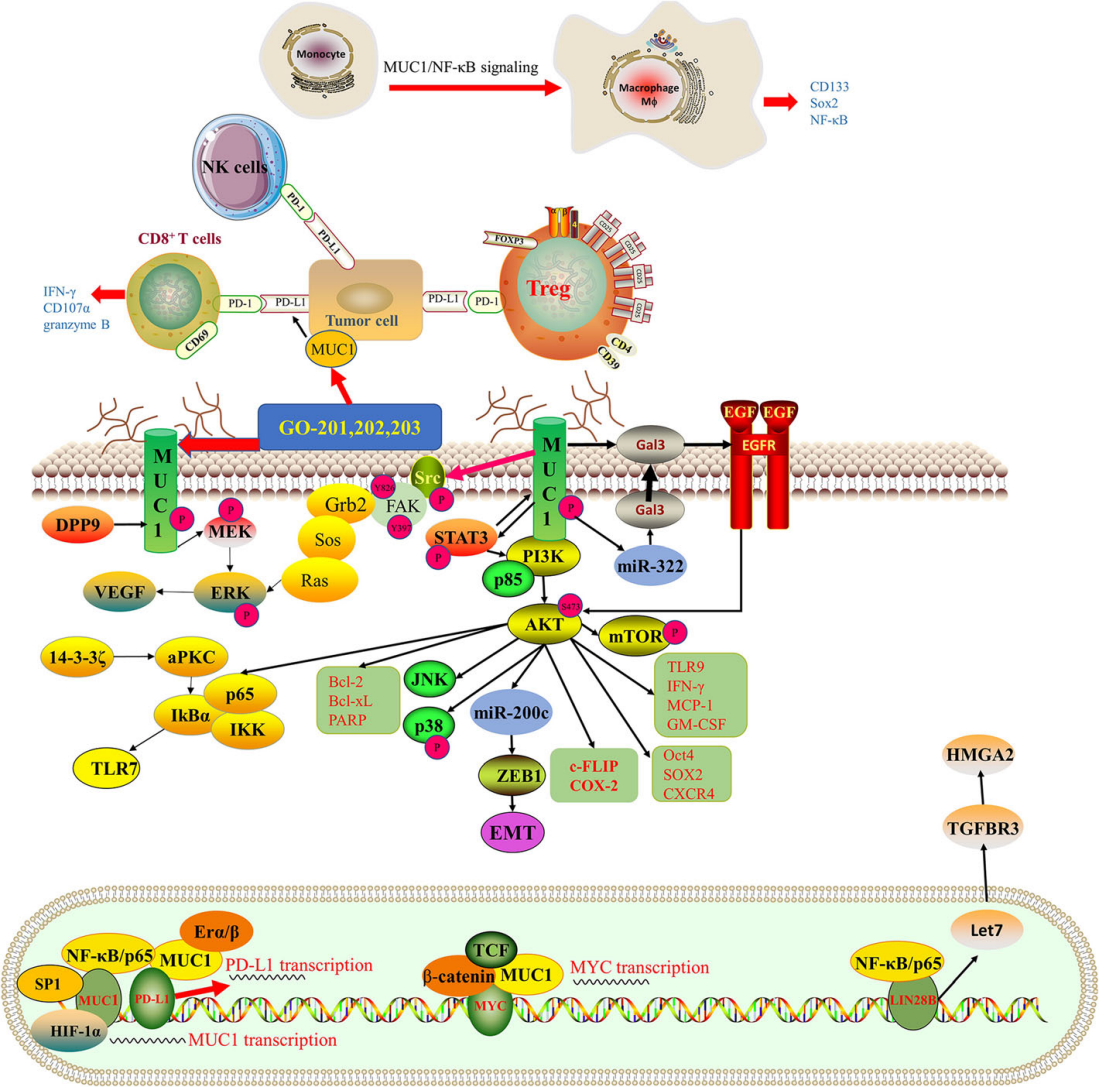
Schematic diagram of pathways MUC1 implicated in non-small cell lung cancer. Aberrantly expression of MUC1 actived the downstream FAK, PI3K/AKT, NF/κB pathways, and MUC1 also implicated in angiogenesis via regulation VEGF expression. Meanwhile, MUC1 participated in the regulation of tumor microenvironment through regulating CD274/PD-L1 expression. Several transcription factors, such as HIF-1α, SP1 and non-coding RNA were invloved in regulating MUC1 expression

The overexpression of MUC1 causes many downstream indications closely related to poor clinical performance (Fig. 2). Giatromanolaki et al. examined the correlation between VEGF and MUC1 expression in 199 NSCLCs, then demonstrated that MUC1 expression is linked to high VEGF expression [20]. And overexpression of MUC1 facilitates angiogenesis of NSCLC by activating the Akt and ERK signaling pathways then up-regulating vascular endothelial growth factor (VEGF) [21]. Gao et al. demonstrated that knockdown MUC1 could activate apoptosis and inhibit cell proliferation and metastasis, as well as be sensitized to cisplatin treatment by modulating STAT3/Akt, SRC/FAK and Bcl-XL/Bcl-2 signaling pathways in NSCLC [22]. Besides, MUC1 could interact with ERα and ERβ within the nucleus of to inhibit the proliferation of LUAD cells [23]. MUC1 is also involved in the NF-κB signaling pathways by forming a complex with NF-κB/p65. The complex is directly brought to the promoter of CD274 driving PD-L1 transcription [24]. Another complex, MUC1/β-catenin/TCF4 is directly bound to the MYC promoter and promotes the recruitment of p300 histone acetylase (EP300), which can induce histone H3 acetylation and MYC gene transcription, in turns downregulate MYC-target genes [25]. MUC1-C induces NF-κB/p65 chromatin occupancy of the LIN28B first intron and activates LIN28B transcription, consequently activates the LIN28B → let-7 → HMGA2 ceRNA axis in NSCLC, and thereby promotes EMT and stemness phenotype [26]. The N-glycosylated MUC1-C restrains miR-322 expression and thereby up-regulates galectin-3. Successively, galectin-3 forms a bridge between MUC1 and the EGFR which physically integrates MUC1 with EGFR signaling [27]. Moreover, MUC1 plays a great role in acquired chemoresistance. In the study of Xu et al. demonstrated that knockout MUC1 could significantly increase the apophatic toxicity of displaying, doxorubicin and TRAIL induced anti-apoptotic lung cancer cells. And miR-551b/catalase/ROS axis gives rise to MUC1 overexposure following EGFR-mediated activation of the cell survival cascade involving Akt/c-FLIP/COX-2 [28]. In PTX-resistant lung cancer cells, overexposure of MUC1 promotes proliferation, stemness by regulating PI3K/Akt signaling and cancer stemness biomarkers [29]. Similarly, MUC4 drops lung cancer cells proliferation through down-regulating of cell cycle related protein and GSK3β/p-Akt, which regulates the invasion and metastasis by FAK activity and EMT marker [30]. MUC5AC interacts with integrin β4 recruit phosphorylation of FAK (Y397) activated downstream signaling pathways, leading to lung cancer cell migration [31]. Wei Han et al. demonstrated knockdown MUC5AC could significantly downregulate PCNA which is a well-known proliferation biomarker, and metastasis biomarker MMP-2, MMP-9 [15]. MUC16 could promote lung cancer progression, metastasis, and chemoresistance to cisplatin and gemcitabine via the regulation of TSPYL5 activity through JAK2/STAT3/GR axis [32]. MUC16 mutations are associated with MUC16 mRNA and protein up-regulation, furthermore promotes the proliferation, enhances migration and invasion and increases cisplatin resistance of lung cancer [33, 34].

### Regulation of the expression of Mucin family genes

**Fig. 3:**
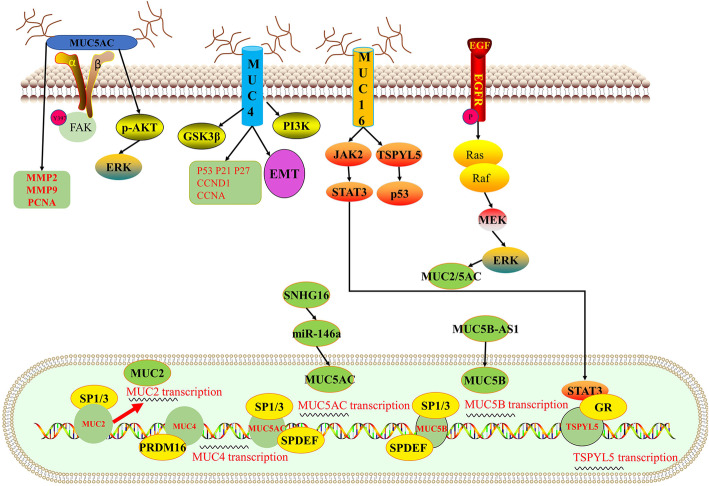
Schematic diagram of signaling pathways other mucins involved in non-small cell lung cancer. MUC5AC implicated in activating FAK and AKT pathways. MUC4 promote the proliferation and metastasis of lung cancer through regulation cell cycle related genes and EMT. MUC16 regulated the transcription of TSPYL5 via JAK/STAT3 and P53 pathways. Moreover, EGFR regulated the MUC2/5 AC expression through ERK pathway

There are still various transcription factors and signaling molecules regulating MUC1 gene expression in airway epithelial cells and lung cancer cells (Fig. 3). Sp-1 has been demonstrated to modulate MUC1 expression by being peculiarly binding on the MUC1 promoter between − 99/− 90 in lung cancer cells [35]. Hypoxia actives the HIF-1α interacted with MUC1 promoter then enhances MUC1 expression [36]. The downregulation of 14–3-3ζ could completely clear up the carcinogenic potential of MUC1 through MUC1/NF-κB feedback loop [37]. Fuzhengkangai decoction regulates MUC1 expression through Akt-mediated inhibition of p65 [38]. Besides, STAT3 and DPP9 are two upstream regulators of MUC1 which can regulate MUC1 expression at both mRNA and protein levels [22, 39]. EGF and TGF-α induces MUC2 and MUC5AC expression through EGFR/Ras/Raf/ERK-signaling Cascade. In addition, Sp-1 and Sp-3 regulates MUC2 and MUC5AC expression by binding their promoters [40]. PRDM16-ΔPRD regulates transcription of MUC4 by regulating the histone modifications of its promoter [41]. SPDEF regulates the expression of MUC5AC and MUC5B combining with the upstream enhancer regions of the MUC5AC and MUC5B [42]. Besides, two long non-coding RNA have been reported involved in regulating mucins. SNHG16-miR-146a axis stimulate MUC5AC expression in NSCLC [15]. MUC5B-AS1, as a novel long non-coding antisense transcript, promotes cell migration and invasion by forming a RNA-RNA duplex with MUC5B, thereby increases MUC5B expression levels in lung adenocarcinoma [43].

### The importance of Mucin for the tumor immune microenvironment

**Fig. 4:**
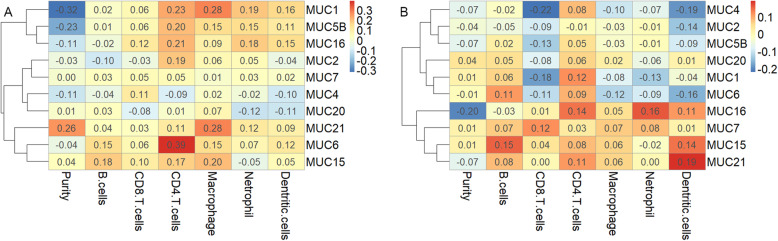
The relation between mucins mRNA expression and tumor-immune infiltrating (TICs) (B-cells, CD4+ T-cells, CD8+ T-cells, neutrophils, macrophages and dendritic cells). **a**. The correlation between each type of TICs and mucins in LUAD, MUC6 indicated the highest positively correlation with CD4+ T cells infiltrating, while MUC1 and MUC21 showed higher correlation with macrophages. **b**. The correlation between each type of TICs and mucins in LUSC, MUC4 showed a higher correlation with CD8+ T cells, while MUC4 and MUC21 both showed higher correlation with dendritic cells

Recently, cancer immune microenvironment has proved of great significance for immunotherapy. Several studies have reported that evaluating MUC1 in tumor cells is relating to the evasion of immune recognition and destruction in NSCLC (Fig. 2). MUC1 plays a key role in TAM-induced in the generation of lung cancer stem cells (LCSCs) progression by regulating NF-κB, CD133, and Sox2 [44]. Targeting MUC1-C drives the aberrant downregulation of PD-L1, IFN-γ and leads to enhanced effector function of CD8+ tumor-infiltrating lymphocytes (TILs) in the tumor microenvironment [45]. Knockdown MUC1-C inhibits PD-L1 and TLR9, IFN-γ, MCP-1 and GM-CSF expression in NSCLC tumors [24]. Furthermore, the connection between the immune infiltration levels and the expression of mucins in LUAD and LUSC patients was discussed based on TIMER (Fig. 4a-b) [46, 47]. MUC1 mRNA expression was significantly positively correlated with infiltrating levels of macrophages, Netrophil and Dentritic cells, and MUC6 mRNA expression level showed a significantly positively connection with infiltrating levels of CD4+ T cells in LUSC. In LUAD, MUC16 mRNA expression indicated the association with CD4+ T-cells, Netrophil. MUC4 has a negative correlation with Dentritic Cells and CD8+ T cells. And MUC21 positively correlates with Dentritic Cells.

### Mucins and therapeutic perspectives

Various studies demonstrated that MUC1 plays an important role in drug-resistance, targeted therapy of lung cancer, which makes it an attractive target for lung cancer therapy. The MUC1 inhibitor GO-201, 202 and 203 can bind directly with the cytoplasmic domain of MUC1 thereby weaken MUC1-mediated cell proliferation [48]. And, GO-203 blocks homoerotic dimerization of MUC1-C, and reverses the MUC1 carcinogenic effect in NSCLC [49]. Several studies have reported how G0–203 work in NSCLC. GO-203 inhibits NSCLC cell growth and survival by preventing the integration between MUC1-C and PI3K-p85, and suppresses constitutive phosphorylation of Akt and its downstream effector, mTOR [48]. Furthermore, Go-203 also plays an important role in the regulation of EGFR-TKI resistance treatment. Silencing MUC1-C in H1975/EGFR(L858R/T790M) cells suppresses AKT signaling pathway, and inhibits cell proliferation of lung cancer [50]. Combining GO-203 with afatinib work synergistically can inhibit the growth of NSCLC cells with EGFR(T790M) or EGFR (delE746-A750) mutants [50]. Combining GO-203 with JQ1 which mechanically inhibits MYC expression shows synergistic function in inhibiting growth of NSCLC tumor xenografts [25]. Silencing MUC1-C in KRAS(G12S) and KRAS(Q61H) mutated NSCLC cells results in downregulation of AKT and MEK signaling and represses ZEB1/miR-200c loop, thereby reverses the EMT phenotype, decreases self-renewal and attenuates the proliferation of KRAS mutant NSCLC cells [51]. Most of all, treatment with GO-203 destroy the MUC1-C → PD-L1 signaling, and promotes the suppression of CD8+ T cell activation [45]. Integrating GO-203 with immune checkpoint inhibitors may be a potential approach for NSCLC therapy.

MUC1 also serves as TAAs playing an important role in tumor immunotherapy. There are two vaccines for NSCLC targeted MUC1 being in clinical trials. TG4010 is an immunotherapeutic vaccine based on Modified Vaccinia virus Ankara (MVA), and encoding the human tumor-associated antigen MUC1 and human IL-2. In Phase II study of TG4010, there were 65 patients with MUC1 positively treated with TG4010 in combination with cisplatin and vinorelbine as first-line chemotherapy. The 65 patients were divided into two groups: Group 1, a TG4010-chemotherapy combination; and Group 2, a sequential protocol in which TG4010 was first administered as monotherapy until got partial response then combined with chemotherapy. The median overall survival (OS) was 12.7 months and 14.9 months respectively [52]. In the study of Quoix et al. (NCT00415818), 148 patients with advanced (stage IIIB or IV) NSCLC with MUC1 positively were enrolled in parallel groups, that patients in experiment treated were allocated to the combination therapy group, and received TG4010 plaque forming with TG4010 plus cisplatin and gemcitabine while the control group received the same chemotherapy alone. The 6-month progression-free survival (PFS) was 43.2% in the TG4010 plus chemotherapy group, and 35.1% in the chemotherapy alone group [53]. In another study of Quoix et al. (NCT01383148), they recruited 222 patients and randomly allocated averagely into TG4010 and chemotherapy, placebo and chemotherapy 111 groups. The results indicated that median PFS was 5.9 months in the TG4010 group and 5.1 months in the placebo group [54]. Both of these studies demonstrated TG4010 plus chemotherapy improve PFS and OS outcome in MUC1-positive patients. Recently, a study of 78 patients which all coming from the TIME study carrying the HLA-A02*01 haplotype indicated TG4010 treatment broadens CD8 + T cell against responses to MUC1 as well as other non-targeted TAA [55]. Therefore, TG4010 can be used in combination with other targeted immunomodulators to maximize response rates and clinical benefits. Sequential treatment with anti-PD-1/PD-L1 after treated with TG4010(NCT02823990) shows a better overall survival in mice model [56]. Moreover, there are two clinical trials (NCT02823990 and NCT03353675) in studying about combing TG4010 and Nivolumab in NSCLC patients (Table 2).

**Table 2:** Current recruiting and non-recruiting clinical trials of mucins

Drug Name	Clinical trial ID	Phase	Clinical trial
TG4010+ chemotherapy	NCT00415818	Phase 2/3	A Phase IIb Multicentric Controlled Study Evaluating the Therapeutic Vaccine TG4010(MVA-MUC1-IL2) as an Adjunct to Standard Chemotherapy in Advanced Non-Small Cell Lung Cancer
TG4010 + placebo	NCT01383148	Phase 2/3	A Phase IIB/III Randomized, Double-blind, Placebo Controlled Study Comparing First Line Therapy with or Without TG4010 Immunotherapy Product in Patients with Stage IV NSCLC
TG4010 + Nivolumab	NCT02823990	Phase 2	Phase II Trial of TG4010 Plus Nivolumab in Previously Treated Patients with Metastatic NSCLC
TG4010 + Nivolumab	NCT03353675	Phase 2	A Phase II Study Evaluating the Efficacy and the Safety of First-line Chemotherapy Combined with TG4010 and Nivolumab in Patients with Advanced Non-squamous NSCLC
Tecemotide (L-BLP25)	NCT00157196	Phase 2	A Multi-center, Non-randomized, Open Label Safety Study of BLP25 Liposome Vaccine (L-BLP25) in NSCLC Patients with Unresectable Stage III Disease
Tecemotide (L-BLP25)	NCT00157209	Phase 2	A Multicenter Phase IIb Randomised, Controlled Study of BLP25 Liposome Vaccine for Active Specific Immunotherapy of NSCLC
Tecemotide	NCT00409188	Phase 3	A Multi-center Phase III Randomized, Double-blind Placebo-controlled Study of the Cancer Vaccine Stimuvax® (L-BLP25 or BLP25 Liposome Vaccine) in NSCLC Subjects with Unresectable Stage III Disease.
Tecemotide (L-BLP25)	NCT01015443	Phase 3	A Multi-national, Double-blind, Placebo-controlled, Randomized, Phase III Clinical Trial of the Cancer Vaccine Stimuvax® (L-BLP25 or BLP25 Liposome Vaccine) in Asian Subjects with Stage III, Unresectable, NSCLC Who Have Demonstrated Either Stable Disease or Objective Response Following Primary Chemo-radiotherapy
Tecemotide	NCT02049151	Phase 3	A Multicenter, Randomized, Double-blind, Placebo-controlled Phase III Trial of Tecemotide Versus Placebo in Subjects with Completed Concurrent Chemo-radiotherapy for Unresectable Stage III NSCLC
Tecemotide	NCT00960115	Phase 1/2	Combined Phase I/II Clinical Study of EMD531444(L-BLP25 or BLP25 Liposome Vaccine) in Subjects with Stage III Unresectable Non-Small Cell Lung Cancer Following Primary Chemoradiotherapy
Tecemotide+Bevacizumab	NCT00828009	Phase 2	A Phase II Study of L-BLP25 and Bevacizumab in Unresectable Stage IIIA and IIIB Non-Squamous Non-Small Cell Lung Cancer After Definitive Chemoradiation
Tecemotide	NCT01423760	Not Applicable	An Open-label Trial to Collect Long-term Data on Subjects Following Participation in Previous EMD 531444 (L-BLP25 or BLP25 Liposome Vaccine) Clinical Trials
ETBX-051+ ETBX-061+ ETBX-011	NCT02140996	Phase 1	Multi-Targeted Recombinant Ad5 (CEA/MUC1/Brachyury) Based Immunotherapy Vaccine Regimen in People with Advanced Cancer
anti-MUC1 CAR T cells	NCT03525782	Phase 1/2	A Clinical Study of Anti-MUC1 CAR T Cells and PD-1 Knockout Engineered T Cells for Patients with Advanced Non-small Cell Lung Cancer
anti-MUC1 CAR-pNK cells	NCT02839954	Phase 1/2	Study Evaluating the Efficacy and Safety of Chimeric Antigen Receptor-Modified pNK Cells in MUC1 Positive Advanced Refractory or Relapsed Solid Tumor
anti-MUC1 CAR T cells	NCT02587689	Phase 1/2	Phase I/II Study of Anti-MUC1 CAR T Cells for Patients with MUC1+ Advanced Refractory Solid Tumor
CART-TnMUC1–01	NCT04025216	Phase 1	A Study of CART-TnMUC1 in Patients with TnMUC1-Positive Advanced Cancers
MUC1 Peptide-Poly-ICLC	NCT03300817	Phase 1	A Pilot Study of MUC1 Vaccine in Current and Former Smokers at High Risk for Lung Cancer
MUC1 peptide-poly-ICLC	NCT01720836	Phase 1/2	Vaccine Therapy in Treating Patients with Stage I-III Non-Small Cell Lung Cancer

Tecemotide, also known as L-BLP25 or Stimuvax, is designed to elicit an antigen-specific cellular immune response against MUC1, which is one of the first TAAs identified by human tumor-specific T-cells. Palmer M et al. performed a phase 1 study of L-BLP25 in patients with stage IIIB or IV NSCLC, which certified that L-BLP25 were well tolerated for patients [57]. Later, Charles Butts et al. conducted a Phase IIB Trial in stage IIIB or IV NSCLC patients, which patients were treated with either L-BLP25 plus best supportive care (BSC) or BSC alone. The 3-year following up results demonstrated a median survival time was longer in patients treated with L-BLP25 plus BSC compared with BSC alone, and patients in stage IIIB LR disease showed the greatest difference [58, 59]. The phase 3 Trial of L-BLP25, which recruited 1513 patients with Stage III NSCLC (NCT00409188), the results demonstrated that patients treated with L-BLP25 have longer median OS (25.6 months) versus placebo (22.3 months), combined L-BLP25 with chemoradiotherapy had a markedly longer median OS (30.8 months) than placebo (20.6 months) while sequential chemoradiotherapy with L-BLP25 (19.4 months) shows no difference versus placebo (24.6 months) [60]. Conversely, Phase I/II Study of Nobuyuki et al. in Japanese unresectable Stage III NSCLC (NCT00960115) found that L-BLP25 has no greater treatment effect in individuals than those received primary concurrent chemoradiotherapy [61]. However, there are still various clinical trials of L-BLP25 under study about CAR T cells therapy and Vaccine for prevention for lung cancer (Table 2).

## Conclusions

Although mucins in lung cancer are not well studied because of its heavy molecular weight, they are still tended to play a significant role in lung carcinogenesis. Mucins served as an important diagnostic method is widely used in clinical especially MUC1 and MUC16 due to their unique expression pattern and function. And their therapeutic potential in lung cancer deserve further studies. And the association between different Mucins will make a specific degree of sophistication in our understanding to their function in lung carcinogenesis.

MUC1-targeted vaccines and small molecule drugs are now in clinical studies for preventing lung cancer. However, the effect of those vaccines was rarely as expected, which makes it necessary to develop new drugs for MUC1 or other mucins. Moreover, it seems that MUC16, MUC21 and MUC5B showed high mutation rates, mRNA expression and close relations to tumor immune infiltration may be still a great target for lung cancer target and immune therapy.

In addition, further researches about the role of mucins in lung cancer with different mutational background such as K-ras, EGFR, and BRAF are necessary to guide the combination therapy and overcome drug-resistance for lung cancer.

## Data Availability

The datasets used during the present study are available from the corresponding author upon reasonable request. Data were obtained from The Cancer Genome Atlas (TCGA; https://portal.gdc.cancer.gov), cBioportal (https://www.cbioportal.org/), the University of California Santa Cruz Xena Browser (https://xenabrowser.net), and TIMER (http://timer.cistrome.org/).

## Abbreviations

MSKCC: Memorial sloan kettering cancer center
GTEx: Genotype-tissue expression
TCGA: The cancer genome atlas
VEGF: Vascular endothelial growth factor
AKT: Protein kinase B
STAT3: Signal transducer and activator of transcription 3
ERK1/2: Extracellular regulated protein kinases1/2
SRC: Src family kinases
FAK: Focal adhesion kinase
Bcl-xL: B-cell lymphoma-extra Large
Bcl-2: B-cell lymphoma-2
ERα: Estrogen receptor α
ERβ: Estrogen receptor β
NF-κB: Nuclear factor-kappa B
CD274/PD-L1: Programmed cell death 1 ligand 1
TCF4: Transcription factor 4
EP300: Histone acetyltransferase p300
HMGA2: High mobility group protein HMGI-C
EMT: Epithelial–mesenchyme transition
EGFR: Epidermal growth factor receptor
ROS: Reactive oxygen species
c-FLIP: Cellular FLICE-like inhibitory protein
COX-2: Cyclooxygenase-2
GSK3β: Glycogen synthase kinase-3β
p-Akt: Phosphorylated RAC-alpha serine/threonine-protein kinase
PCNA: Proliferating cell nuclear antigen
MMP2: Matrix metalloproteinase-2
MMP-9: Matrix metalloproteinase-9
TSPYL5: Testis-specific Y-encoded-like protein 5
JAK2: Janus kinase 2
DPP9: Dipeptidyl peptidase 9
EGF: Epidermal growth factor
TGF-β1: Transforming growth factor
RAS: Renin-angiotensin system
Raf: Rapidly accelerated fibrosarcoma
SP1/3: Specificity protein-1/3
PRDM16: PR domain-containing protein 16
SPDEF: SAM-pointed domain-containing Ets-like factor
IFN-γ: Interferon-gamma
TILs: Tumor-infiltrating lymphocytes
TLR9: Toll-like receptor 9
MCP-1: Monocyte chemoattractant protein-1.
GM-CSF: Granulocyte-macrophage colony-stimulating factor
mTOR: Mammalian/mechanistic target of rapamycin
EGFR-TKI: Epidermal growth factor receptor tyrosine kinases inhibitors
KRAS: Kirsten-RAS
ZEB1: Zinc finger E-box binding homeobox 1
IL-2: Interleukin-2
TAA: Tumor-associated antigens

## References

[1] M. Rebecca L. Siegel, M. Kimberly D. Miller, and D. P. Ahmedin Jemal, "Cancer Statistics, 2019,".2019). 10.3322/caac.21551 30620402

[2] Molina JR, Yang P, Cassivi SD, Schild SE, Adjei AA (2008). Non-small cell lung Cancer: epidemiology, risk factors, treatment, and survivorship. Mayo Clin Proc.

[3] American Cancer Society (2019). Cancer Facts & Figures 2019.

[4] Herbst RS, Morgensztern D, Boshoff C (2018). The biology and management of non-small cell lung cancer. Nature..

[5] Kufe DW (2009). Mucins in cancer: function, prognosis and therapy. Nat Rev Cancer.

[6] Xu M, Wang DC, Wang X, Zhang Y (2017). Correlation between mucin biology and tumor heterogeneity in lung cancer. Semin Cell Dev Biol.

[7] Lakshmanan I, Ponnusamy MP, Macha MA, Haridas D, Majhi PD, Kaur S, Jain M, Batra SK, Ganti AK (2015). Mucins in lung cancer: diagnostic, prognostic, and therapeutic implications. J Thorac Oncol.

[8] Goldman MJ, Craft B, Hastie M, Repecka K, McDade F, Kamath A, Banerjee A, Luo Y, Rogers D, Brooks AN, Zhu J, Haussler D (2020). Visualizing and interpreting cancer genomics data via the Xena platform. Nat Biotechnol.

[9] Consortium GT. The genotype-tissue expression (GTEx) project. Nat Genet. 2013;45:580–5. 10.1038/ng.2653 PMC4010069 23715323

[10] J. Lonsdale, J. Thomas, M. Salvatore, R. Phillips, E. Lo, S. Shad, R. Hasz, G. Walters, F. Garcia, N. Young, B. Foster, M. Moser, E. Karasik, B. Gillard, K. Ramsey, S. Sullivan, J. Bridge, H. Magazine, J. Syron, J. Fleming, L. Siminoff, H. Traino, M. Mosavel, L. Barker, S. Jewell, D. Rohrer, D. Maxim, D. Filkins, P. Harbach, E. Cortadillo, B. Berghuis, L. Turner, E. Hudson, K. Feenstra, L. Sobin, J. Robb, P. Branton, G. Korzeniewski, C. Shive, D. Tabor, L. Qi, K. Groch, S. Nampally, S. Buia, A. Zimmerman, A. Smith, R. Burges, K. Robinson, K. Valentino, D. Bradbury, M. Cosentino, N. Diaz-Mayoral, M. Kennedy, T. Engel, P. Williams, K. Erickson, K. Ardlie, W. Winckler, G. Getz, D. DeLuca, D. MacArthur, M. Kellis, A. Thomson, T. Young, E. Gelfand, M. Donovan, Y. Meng, G. Grant, D. Mash, Y. Marcus, M. Basile, J. Liu, J. Zhu, Z. Tu, N. J. Cox, D. L. Nicolae, E. R. Gamazon, H. K. Im, A. Konkashbaev, J. Pritchard, M. Stevens, T. Flutre, X. Wen, E. T. Dermitzakis, T. Lappalainen, R. Guigo, J. Monlong, M. Sammeth, D. Koller, A. Battle, S. Mostafavi, M. McCarthy, M. Rivas, J. Maller, I. Rusyn, A. Nobel, F. Wright, A. Shabalin, M. Feolo, N. Sharopova, A. Sturcke, J. Paschal, J. M. Anderson, E. L. Wilder, L. K. Derr, E. D. Green, J. P. Struewing, G. Temple, S. Volpi, J. T. Boyer, E. J. Thomson, M. S. Guyer, C. Ng, A. Abdallah, D. Colantuoni, T. R. Insel, S. E. Koester, A. R. Little, P. K. Bender, T. Lehner, Y. Yao, C. C. Compton, J. B. Vaught, S. Sawyer, N. C. Lockhart, J. Demchok, and H. F. Moore, "The Genotype-Tissue Expression (GTEx) project," Nat Genet. 45, 580–585.(2013). 10.1038/ng.2653 PMC4010069 23715323

[11] Lappi-Blanco E, Mäkinen JM, Lehtonen S, Karvonen H, Sormunen R, Laitakari K, Johnson S, Mäkitaro R, Bloigu R, Kaarteenaho R (2016). Mucin-1 correlates with survival, smoking status, and growth patterns in lung adenocarcinoma. Tumor Biol.

[12] Guddo F, Giatromanolaki A, Koukourakis MI, Reina C, Vignola AM, Chlouverakis G, Hilkens J, Gatter KC, Harris AL, Bonsignore G (1998). MUC1 (episialin) expression in non-small cell lung cancer is independent of EGFR and c-erbB-2 expression and correlates with poor survival in node positive patients. J Clin Pathol.

[13] Woenckhaus M, Merk J, Stoehr R, Schaeper F, Gaumann A, Wiebe K, Hartmann A, Hofstaedter F, Dietmaier W (2008). Prognostic value of FHIT, CTNNB1, and MUC1 expression in non-–small cell lung cancer. Hum Pathol.

[14] Yu CJ, Shih JY, Lee YC, Shun CT, Yuan A, Yang PC (2005). Sialyl Lewis antigens: association with MUC5AC protein and correlation with post-operative recurrence of non-small cell lung cancer. Lung Cancer.

[15] Han W, Du X, Liu M, Wang J, Sun L, Li Y (2019). Increased expression of long non-coding RNA SNHG16 correlates with tumor progression and poor prognosis in non-small cell lung cancer. Int J Biol Macromol.

[16] Situ D, Wang J, Ma Y, Zhu Z, Hu Y, Long H, Rong T (2011). Expression and prognostic relevance of MUC1 in stage IB non-small cell lung cancer. Med Oncol.

[17] Cerami E, Gao J, Dogrusoz U, Gross BE, Sumer SO, Aksoy BA, Jacobsen A, Byrne CJ, Heuer ML, Larsson E, Antipin Y, Reva B, Goldberg AP, Sander C, Schultz N (2012). The cBio Cancer genomics portal: an open platform for exploring multidimensional Cancer genomics data. Cancer Discov.

[18] Gao J, Aksoy BA, Dogrusoz U, Dresdner G, Gross B, Sumer SO, Sun Y, Jacobsen A, Sinha R, Larsson E, Cerami E, Sander C, Schultz N (2013). Integrative analysis of complex cancer genomics and clinical profiles using the cBioPortal. Sci Signal.

[19] Skoulidis F, Heymach JV (2019). Co-occurring genomic alterations in non-small-cell lung cancer biology and therapy. Nat Rev Cancer.

[20] Giatromanolaki A, Koukourakis MI, Sivridis E, O'Byrne K, Cox G, Thorpe PE, Gatter KC, Harris AL (2000). Coexpression of MUC1 glycoprotein with multiple angiogenic factors in non-small cell lung cancer suggests coactivation of angiogenic and migration pathways. Clin Cancer Res.

[21] Yao M, Zhang W, Zhang Q, Xing L, Xu A, Liu Q, Cui B (2011). Overexpression of MUC1 enhances proangiogenic activity of non-small-cell lung cancer cells through activation of Akt and extracellular signal-regulated kinase pathways. Lung..

[22] Gao J, McConnell MJ, Yu B, Li J, Balko JM, Black EP, Johnson JO, Lloyd MC, Altiok S, Haura EB (2009). MUC1 is a downstream target of STAT3 and regulates lung cancer cell survival and invasion. Int J Oncol.

[23] Klinge CM, Radde BN, Imbert-Fernandez Y, Teng Y, Ivanova MM, Abner SM, Martin AL (2011). Targeting the intracellular MUC1 C-terminal domain inhibits proliferation and estrogen receptor transcriptional activity in lung adenocarcinoma cells. Mol Cancer Ther.

[24] Bouillez A, Rajabi H, Jin C, Samur M, Tagde A, Alam M, Hiraki M, Maeda T, Hu X, Adeegbe D, Kharbanda S, Wong KK, Kufe D (2017). MUC1-C integrates PD-L1 induction with repression of immune effectors in non-small-cell lung cancer. Oncogene..

[25] Bouillez A, Rajabi H, Pitroda S, Jin C, Alam M, Kharbanda A, Tagde A, Wong KK, Kufe D (2016). Inhibition of MUC1-C suppresses MYC expression and attenuates malignant growth in KRAS mutant lung adenocarcinomas. Cancer Res.

[26] Alam M, Ahmad R, Rajabi H, Kufe D (2015). MUC1-C induces the LIN28B-->LET-7-->HMGA2 Axis to regulate self-renewal in NSCLC. Mol Cancer Res.

[27] Ramasamy S, Duraisamy S, Barbashov S, Kawano T, Kharbanda S, Kufe D (2007). The MUC1 and Galectin-3 Oncoproteins function in a MicroRNA-dependent regulatory loop. Mol Cell.

[28] Xu X, Wells A, Padilla MT, Kato K, Kim KC, Lin Y (2014). A signaling pathway consisting of miR-551b, catalase and MUC1 contributes to acquired apoptosis resistance and chemoresistance. Carcinogenesis..

[29] Ham SY, Kwon T, Bak Y, Yu JH, Hong J, Lee SK, Yu DY, Yoon DY (2016). Mucin 1-mediated chemo-resistance in lung cancer cells. Oncogenesis.

[30] Majhi PD, Lakshmanan I, Ponnusamy MP, Jain M, Das S, Kaur S, Shimizu ST, West WW, Johansson SL, Smith LM, Yu F, Rolle CE, Sharma P, Carey GB, Batra SK, Ganti AK (2013). Pathobiological implications of MUC4 in non-small-cell lung cancer. J Thorac Oncol.

[31] Lakshmanan I, Rachagani S, Hauke R, Krishn SR, Paknikar S, Seshacharyulu P, Karmakar S, Nimmakayala RK, Kaushik G, Johansson SL, Carey GB, Ponnusamy MP, Kaur S, Batra SK, Ganti AK (2016). MUC5AC interactions with integrin beta4 enhances the migration of lung cancer cells through FAK signaling. Oncogene.

[32] Lakshmanan I, Salfity S, Seshacharyulu P, Rachagani S, Thomas A, Das S, Majhi PD, Nimmakayala RK, Vengoji R, Lele SM, Ponnusamy MP, Batra SK, Ganti AK (2017). MUC16 regulates TSPYL5 for lung Cancer cell growth and Chemoresistance by suppressing p53. Clin Cancer Res.

[33] Patel JS, Callahan BM, Chobrutskiy BI, Blanck G (2019). Matrix-metalloprotease resistant mucin-16 (MUC16) peptide mutants represent a worse lung adenocarcinoma outcome. Proteomics Clin Appl.

[34] Kanwal M, Ding XJ, Song X, Zhou GB, Cao Y (2018). MUC16 overexpression induced by gene mutations promotes lung cancer cell growth and invasion. Oncotarget..

[35] Kuwahara I, Lillehoj EP, Hisatsune A, Lu W, Isohama Y, Miyata T, Kim KC (2005). Neutrophil elastase stimulates MUC1 gene expression through increased Sp1 binding to theMUC1 promoter. Am J Physiol-Lung C.

[36] Mikami Y, Hisatsune A, Tashiro T, Isohama Y, Katsuki H (2009). Hypoxia enhances MUC1 expression in a lung adenocarcinoma cell line. Biochem Bioph Res Co.

[37] Xue M, Tao W (2017). Upregulation of MUC1 by its novel activator 14-3-3zeta promotes tumor invasion and indicates poor prognosis in lung adenocarcinoma. Oncol Rep.

[38] Li L, Wang S, Zheng F, Wu W, Hann SS (2016). Chinese herbal medicine Fuzheng Kang-Ai decoction sensitized the effect of gefitinib on inhibition of human lung cancer cells through inactivating PI3-K/Akt -mediated suppressing MUC1 expression. J Ethnopharmacol.

[39] Tang Z, Li J, Shen Q, Feng J, Liu H, Wang W, Xu L, Shi G, Ye X, Ge M, Zhou X, Ni S (2017). Contribution of upregulated dipeptidyl peptidase 9 (DPP9) in promoting tumoregenicity, metastasis and the prediction of poor prognosis in non-small cell lung cancer (NSCLC). Int J Cancer.

[40] Perrais M, Pigny P, Copin MC, Aubert JP, Van Seuningen I (2002). Induction of MUC2 and MUC5AC mucins by factors of the epidermal growth factor (EGF) family is mediated by EGF receptor/Ras/Raf/extracellular signal-regulated kinase cascade and Sp1. J Biol Chem.

[41] Fei LR, Huang WJ, Wang Y, Lei L, Li ZH, Zheng YW, Wang Z, Yang MQ, Liu CC, Xu HT (2019). PRDM16 functions as a suppressor of lung adenocarcinoma metastasis. J Exp Clin Cancer Res.

[42] Guo M, Tomoshige K, Meister M, Muley T, Fukazawa T, Tsuchiya T, Karns R, Warth A, Fink-Baldauf IM, Nagayasu T, Naomoto Y, Xu Y, Mall MA, Maeda Y (2017). Gene signature driving invasive mucinous adenocarcinoma of the lung. Embo Mol Med.

[43] Yuan S, Liu Q, Hu Z, Zhou Z, Wang G, Li C, Xie W, Meng G, Xiang Y, Wu N, Wu L, Yu Z, Bai L, Li Y (2018). Long non-coding RNA MUC5B-AS1 promotes metastasis through mutually regulating MUC5B expression in lung adenocarcinoma. Cell Death Dis.

[44] Huang WC, Chan ML, Chen MJ, Tsai TH, Chen YJ (2016). Modulation of macrophage polarization and lung cancer cell stemness by MUC1 and development of a related small-molecule inhibitor pterostilbene. Oncotarget.

[45] Bouillez A, Adeegbe D, Jin C, Hu X, Tagde A, Alam M, Rajabi H, Wong KK, Kufe D (2017). MUC1-C promotes the suppressive immune microenvironment in non-small cell lung cancer. Oncoimmunology.

[46] Li T, Fan J, Wang B, Traugh N, Chen Q, Liu JS, Li B, Liu XS (2017). TIMER: a web server for comprehensive analysis of tumor-infiltrating immune cells. Cancer Res.

[47] Li T, Fu J, Zeng Z, Cohen D, Li J, Chen Q, Li B, Liu XS (2020). TIMER2.0 for analysis of tumor-infiltrating immune cells. Nucleic Acids Res.

[48] Raina D, Kosugi M, Ahmad R, Panchamoorthy G, Rajabi H, Alam M, Shimamura T, Shapiro GI, Supko J, Kharbanda S, Kufe D (2011). Dependence on the MUC1-C oncoprotein in non-small cell lung cancer cells. Mol Cancer Ther.

[49] Raina D, Ahmad R, Rajabi H, Panchamoorthy G, Kharbanda S, Kufe D (2012). Targeting cysteine-mediated dimerization of the MUC1-C oncoprotein in human cancer cells. Int J Oncol.

[50] Kharbanda A, Rajabi H, Jin C, Tchaicha J, Kikuchi E, Wong KK, Kufe D (2014). Targeting the oncogenic MUC1-C protein inhibits mutant EGFR-mediated signaling and survival in non-small cell lung cancer cells. Clin Cancer Res.

[51] Kharbanda A, Rajabi H, Jin C, Alam M, Wong KK, Kufe D (2014). MUC1-C confers EMT and KRAS independence in mutant KRAS lung cancer cells. Oncotarget.

[52] Ramlau R, Quoix E, Rolski J, Pless M, Lena H, Levy E, Krzakowski M, Hess D, Tartour E, Chenard MP, Limacher JM, Bizouarne N, Acres B, Halluard C, Velu T (2008). A phase II study of Tg4010 (Mva-Muc1-Il2) in association with chemotherapy in patients with stage III/IV non-small cell lung cancer. J Thorac Oncol.

[53] Quoix E, Ramlau R, Westeel V, Papai Z, Madroszyk A, Riviere A, Koralewski P, Breton JL, Stoelben E, Braun D, Debieuvre D, Lena H, Buyse M, Chenard MP, Acres B, Lacoste G, Bastien B, Tavernaro A, Bizouarne N, Bonnefoy JY, Limacher JM (2011). Therapeutic vaccination with TG4010 and first-line chemotherapy in advanced non-small-cell lung cancer: a controlled phase 2B trial. Lancet Oncol..

[54] Quoix E, Lena H, Losonczy G, Forget F, Chouaid C, Papai Z, Gervais R, Ottensmeier C, Szczesna A, Kazarnowicz A, Beck JT, Westeel V, Felip E, Debieuvre D, Madroszyk A, Adam J, Lacoste G, Tavernaro A, Bastien B, Halluard C, Palanche T, Limacher JM (2016). TG4010 immunotherapy and first-line chemotherapy for advanced non-small-cell lung cancer (TIME): results from the phase 2b part of a randomised, double-blind, placebo-controlled, phase 2b/3 trial. Lancet Oncol.

[55] Tosch C, Bastien B, Barraud L, Grellier B, Nourtier V, Gantzer M, Limacher JM, Quemeneur E, Bendjama K, Preville X (2017). Viral based vaccine TG4010 induces broadening of specific immune response and improves outcome in advanced NSCLC. J Immunother Cancer.

[56] Remy-Ziller C, Thioudellet C, Hortelano J, Gantzer M, Nourtier V, Claudepierre MC, Sansas B, Preville X, Bendjama K, Quemeneur E, Rittner K (2018). Sequential administration of MVA-based vaccines and PD-1/PD-L1-blocking antibodies confers measurable benefits on tumor growth and survival: preclinical studies with MVA-betaGal and MVA-MUC1 (TG4010) in a murine tumor model. Hum Vaccin Immunother.

[57] M. Palmer, J. Parker, S. Modi, C. Butts, M. Smylie, A. Meikle, M. Kehoe, G. MacLean, and M. Longenecker, "Phase I study of the BLP25 (MUC1 peptide) liposomal vaccine for active specific immunotherapy in stage IIIB/IV non-small-cell lung cancer," Clin Lung Cancer. 3, 49–57, 58.(2001). 10.3816/clc.2001.n.018 14656392

[58] Butts C, Murray N, Maksymiuk A, Goss G, Marshall E, Soulières D, Cormier Y, Ellis P, Price A, Sawhney R, Davis M, Mansi J, Smith C, Vergidis D, Ellis P, MacNeil M, Palmer M (2005). Randomized phase IIB trial of BLP25 liposome vaccine in stage IIIB and IV non–small-cell lung Cancer. J Clin Oncol.

[59] Butts C, Maksymiuk A, Goss G, Soulières D, Marshall E, Cormier Y, Ellis PM, Price A, Sawhney R, Beier F, Falk M, Murray N (2011). Updated survival analysis in patients with stage IIIB or IV non-small-cell lung cancer receiving BLP25 liposome vaccine (L-BLP25): phase IIB randomized, multicenter, open-label trial. J Cancer Res Clin.

[60] Butts C, Socinski MA, Mitchell PL, Thatcher N, Havel L, Krzakowski M, Nawrocki S, Ciuleanu TE, Bosquee L, Trigo JM, Spira A, Tremblay L, Nyman J, Ramlau R, Wickart-Johansson G, Ellis P, Gladkov O, Pereira JR, Eberhardt WE, Helwig C, Schroder A, Shepherd FA (2014). Tecemotide (L-BLP25) versus placebo after chemoradiotherapy for stage III non-small-cell lung cancer (START): a randomised, double-blind, phase 3 trial. Lancet Oncol.

[61] Katakami N, Hida T, Nokihara H, Imamura F, Sakai H, Atagi S, Nishio M, Kashii T, Satouchi M, Helwig C, Watanabe M, Tamura T (2017). Phase I/II study of tecemotide as immunotherapy in Japanese patients with unresectable stage III non-small cell lung cancer. Lung Cancer.

